# Fetal Echocardiography in Gestational Diabetes Mellitus

**DOI:** 10.7759/cureus.89050

**Published:** 2025-07-30

**Authors:** Shaheera Ajaz, Shahood A Kakroo, Huda Amin, Imran Akram, Aamir Rashid

**Affiliations:** 1 Obstetrics and Gynaecology, Directorate of Health Services, Srinagar, IND; 2 Cardiology, Sher-i-Kashmir Institute of Medical Sciences, Srinagar, IND

**Keywords:** diabetes, fetal echocardiography, gestational diabetes mellitus, interventricular septal thickness, pregnancy

## Abstract

Background

Gestational diabetes mellitus (GDM) is associated with significant maternal and fetal complications, including potential effects on fetal cardiac development. This study aimed to evaluate structural and functional cardiac changes in fetuses of mothers with GDM using fetal echocardiography and to compare them with those of healthy pregnancies. The primary objective of this study was to evaluate functional cardiac changes, specifically interventricular septum (IVS) thickness and posterior wall thickness, E/A ratio (ratio of early (E) to late (A) diastolic mitral inflow velocities), and fractional shortening, in fetuses of mothers with GDM. The secondary objective was to assess the presence of structural cardiac abnormalities.

Methodology

This was a comparative, cross-sectional study conducted over six months (January 2024 to July 2024) in the Department of Cardiology and Obstetrics at the Sher-I-Kashmir Institute of Medical Sciences, Srinagar, Jammu and Kashmir, India. The study included 100 women with GDM and 100 with normal pregnancies as controls. Fetal echocardiography was performed between 24 and 28 weeks of gestation using two-dimensional and pulsed-wave Doppler sonography to evaluate structural and functional cardiac parameters. Specifically, measurements included IVS thickness, posterior wall thickness, the E/A ratio, and fractional shortening. These parameters were assessed in fetuses of mothers with GDM and compared with those from healthy pregnancies.

Results

Fetal echocardiographic assessment revealed significantly increased left ventricular posterior wall thickness and IVS thickness in both systole and diastole in the GDM group compared to controls (p < 0.001; 95% confidence interval (CI) = 0.55 to 0.85]. Additionally, the E/A ratio was significantly lower in the GDM group (p < 0.001; 95% CI = -0.06 to -0.01), indicating impaired diastolic function. Despite these changes, no congenital heart defects were identified in either group, as confirmed by postnatal echocardiography.

Conclusions

Fetuses of mothers with GDM exhibit notable structural and functional cardiac changes, including increased myocardial thickness and altered diastolic function. These findings emphasize the importance of fetal echocardiography in pregnancies complicated by GDM to identify early cardiac adaptations and guide clinical management. Further research is needed to explore the long-term cardiovascular implications of these findings.

## Introduction

Gestational diabetes mellitus (GDM) is a common metabolic disorder of pregnancy characterized by glucose intolerance that is first identified during gestation. It affects approximately 7%-18% of pregnancies worldwide, with variations based on population and diagnostic criteria [1]. In India, the prevalence of GDM ranges from 10% to 35%, with higher rates observed in northern states, including Jammu and Kashmir [2]. These variations are influenced by urbanization, dietary patterns, and increasing maternal age. This high regional burden underscores the importance of evaluating fetal outcomes in this population. GDM has significant implications for maternal and fetal health, including increased risks of preeclampsia, macrosomia, and neonatal complications. One of the lesser-studied areas of GDM is its impact on fetal cardiac development, as hyperglycemia can influence fetal organogenesis and growth through mechanisms such as oxidative stress, hyperinsulinemia, and altered placental function [3]. While diabetes mellitus is recognized for its association with an increased risk of cardiovascular abnormalities [3], evidence regarding such risks in individuals with GDM remains limited [4]. In pregnancies complicated by GDM, maternal hyperglycemia leads to fetal hyperinsulinemia, which acts as a fetal growth factor. This condition stimulates cardiomyocyte proliferation and glycogen deposition, particularly in the interventricular septum (IVS) and ventricular walls, resulting in myocardial hypertrophy. Concurrently, oxidative stress and chronic intrauterine inflammation impair fetal myocardial relaxation and contribute to diastolic dysfunction. These pathophysiological changes justify close fetal cardiac monitoring in GDM.

Fetal echocardiography is a powerful, non-invasive diagnostic tool that enables detailed structural and functional assessment of the fetal heart [5,6]. Abnormalities in fetal cardiac dimensions, wall thickness, and function have been observed in pregnancies complicated by GDM, even in cases where postnatal outcomes appear normal. These changes may be subtle and may not manifest as structural congenital heart defects, but could indicate early functional adaptations or remodeling due to maternal hyperglycemia. While structural cardiac anomalies (e.g., hypertrophic cardiomyopathy) have been documented in GDM, evidence remains limited and inconsistent regarding subclinical functional abnormalities, such as diastolic dysfunction, altered myocardial performance index, and long-term cardiovascular programming in offspring. Our study seeks to address this gap by evaluating early functional and structural cardiac parameters via fetal echocardiography.

We hypothesized that fetuses of mothers with GDM would demonstrate subclinical structural or functional cardiac alterations, such as changes in diastolic filling patterns or ventricular wall thickness, compared to fetuses of normoglycemic mothers. In this comparative cross-sectional study, we aimed to investigate the structural and functional cardiac parameters in fetuses of mothers with GDM compared to those of healthy pregnancies. By utilizing fetal echocardiography and Doppler techniques, we sought to elucidate the impact of GDM on fetal cardiac development and identify early markers of potential cardiovascular risk. The primary objective of this study was to evaluate functional cardiac changes, specifically IVS and posterior wall thickness, E/A ratio (ratio of early (E) to late (A) diastolic mitral inflow velocities), and fractional shortening, in fetuses of mothers with GDM. The secondary objective was to assess the presence of structural cardiac abnormalities.

## Materials and methods

Study design

This comparative, cross-sectional study was conducted over six months (January 2024 to July 2024) in the Department of Cardiology and the Department of Obstetrics at the Sher-I-Kashmir Institute of Medical Sciences, Srinagar, Jammu and Kashmir, India. The objective was to evaluate both structural and functional fetal cardiac alterations in pregnancies affected by GDM compared to normal pregnancies.

Inclusion and exclusion criteria

The case group consisted of pregnant women diagnosed with GDM between 24 and 28 weeks of gestation. The control group comprised pregnant women with normoglycemic, uncomplicated pregnancies attending routine prenatal care during the same gestational window (24-28 weeks). Controls were selected using simple random sampling from the antenatal outpatient department during the same study period to ensure comparability. The GDM group underwent fetal echocardiography based on referrals from the Department of Obstetrics due to gestational diabetes. The control group was also recruited through the Department of Obstetrics. These were normoglycemic pregnant women without any known comorbidities, who were referred after obtaining informed consent for participation in the study. Women with pre-existing diabetes mellitus, chronic hypertension, preeclampsia, chronic kidney or liver disease, or connective tissue disorders were excluded from both groups.

GDM diagnosis and management

GDM was diagnosed based on the International Association of the Diabetes and Pregnancy Study Groups criteria [7], using a 75-g oral glucose tolerance test (OGTT), with plasma glucose levels measured at fasting, one-hour, and two-hour intervals. A diagnosis of GDM was made if any one of the following values was met or exceeded: fasting ≥92 mg/dL, one-hour ≥180 mg/dL, or two-hour ≥153 mg/dL. Patients with GDM were managed according to standard institutional protocols. The majority were diet-controlled. Those with persistently elevated glucose levels despite dietary modifications were initiated on insulin therapy. Optimal glycemic control was defined as adherence to the target blood glucose levels recommended by the American Diabetes Association for gestational diabetes, which include a fasting blood glucose level of less than 95 mg/dL, a one-hour postprandial level of less than 140 mg/dL, and a two-hour postprandial level of less than 120 mg/dL.

Echocardiographic assessment

**Figure 1:**
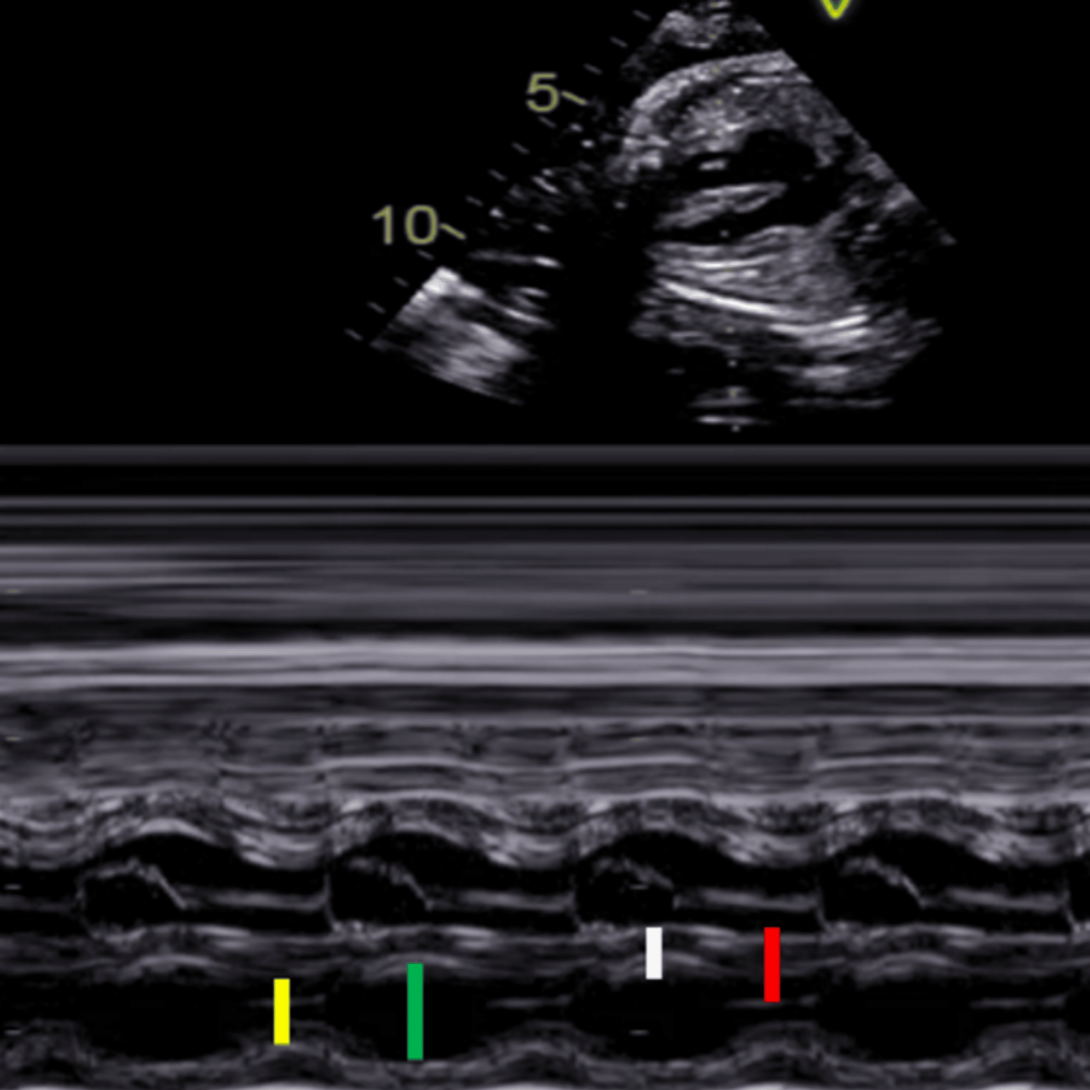
M-mode echocardiographic image obtained from the fetal four-chamber view demonstrating measurement of interventricular septal thickness during diastole (IVSd) and systole (IVSs), as well as left ventricular internal dimensions during diastole (LVIDd) and systole (LVIDs). The white line represents IVSd. The red line shows IVSs. The yellow line marks the LVIDs, and the green line indicates the LVIDd.

Fetal echocardiography was performed by two dedicated pediatric cardiologists on Vivid S5 machines with an M3S matrix array probe with a frequency range from 1.7 to 3.2 MHz (GE Vingmed, Horten, Norway). The procedure was conducted according to the American Society of Echocardiography guidelines [8], with measurements taken between 24 and 28 weeks of gestation. Both assessors were blinded to the maternal GDM status to reduce observer bias. Measurements of the IVS and ventricular wall were obtained from an apical four-chamber view. Three readings were averaged for accuracy. The parameters studied were mitral inflow parameters, including early diastolic peak flow velocity (E) and late diastolic peak flow velocity (A), left ventricular dimensions, and fractional shortening (Figure 1).

The use of percentages to report the mean value was reported to obtain the coefficient of variation, which was calculated for intra-observer and inter-observer variability. The intra-observer coefficient of variation (CV) for IVS was 2.1%. The inter-observer CV for IVS was 3.2%. The intra-observer coefficient of variation (CV) for the ventricular wall was 2.4%. The inter-observer CV for the ventricular wall was 2.2%. The intra-observer CV for fractional shortening was 2.5%. The inter-observer CV for fractional shortening was 3.2%. The CV (both intra- and inter-observer) for other diastolic echocardiographic parameters was less than 5%. Postnatal echocardiographic screening was performed within the first week of life (day three to seven) by pediatric cardiologists using standard protocols.

Sample size

As this was an exploratory study, a formal a priori sample size calculation was not performed. A convenience sample of 100 GDM and 100 control pregnancies was selected based on recruitment feasibility within the defined study window and resource availability.

Statistical analysis

All parameters were recorded in a predefined proforma and analyzed using SPSS software version 19.0 (IBM Corp., Armonk, NY, USA). Continuous variables were expressed as mean ± standard deviation and compared between groups using the independent t-test. Categorical variables were compared using the chi-square test. In addition, multivariate linear regression analysis was performed to adjust for potential confounders such as maternal age, body mass index (BMI), birth weight, parity, and gestational age at delivery, with GDM as the primary independent variable. All statistical tests were two-tailed, and a p-value <0.05 was considered statistically significant.

Ethical considerations

All participants provided written informed consent. The study protocol was approved by the Institutional Ethics Committee of Sher-I-Kashmir Institute of Medical Sciences (approval number: SIMS 41/2025).

## Results

A total of 240 pregnant women were initially screened for eligibility. Of these, 40 women were excluded due to various reasons: 15 did not meet the inclusion criteria, 10 declined to provide informed consent, 8 had incomplete fetal echocardiographic data, and 7 were lost to follow-up. The remaining 200 women were enrolled in the study and divided into the following two groups: 100 women diagnosed with GDM formed the GDM group, while 100 normoglycemic women served as the control (non-GDM) group. All participants delivered during the study period and were followed up for three months postpartum. In the GDM group, 90 patients achieved and maintained optimal glycemic control throughout pregnancy. The mean maternal age was 27.8 ± 4.0 years, with a mean BMI of 27.6 kg/m² at the initial prenatal visit. The glycemic profile of the GDM group (mean ± SD) was as follows: fasting blood glucose, 96.2 ± 8.4 mg/dL; one-hour OGTT, 172.5 ± 14.6 mg/dL; and two-hour OGTT, 153.4 ± 12.1 mg/dL. In comparison, the control group exhibited significantly lower glycemic values: fasting blood glucose, 82.4 ± 6.1 mg/dL; one-hour OGTT, 128.6 ± 10.8 mg/dL; two-hour OGTT, 116.9 ± 9.7mg/dL; and HbA1c, 5.1 ± 0.2%. Fetal echocardiography was performed between 24 and 28 weeks of gestation. Table 1 presents the demographic details.

**Table 1:** Demographic characteristics of women with normal pregnancies and gestational diabetes mellitus.

Characteristic	Normal pregnancies (n = 100)	Gestational diabetes (n = 100)	P-value	t/chi-square value	Test used
Maternal age, years	26.2 ± 4.3	27.9 ± 4.0	0.13	-2.89	Independent t-test
Body mass index, kg/m²	27.0 (23–36)	28.3 (23–38)	0.24	-1.85	Independent t-test
Birth weight, kg	2.8 ± 0.23	3.1 ± 0.32	0.11	-7.61	Independent t-test
Primipara (%)	76 (76%)	75 (75%)	0.44	0.00	Chi-square
Gestational age at delivery, weeks	38.2 ± 1.1	38.3 ± 1.2	0.24	-1.1	Independent t-test

Measurements of cardiac dimensions, including fetal left ventricular posterior wall thickness and IVS thickness at both end-diastole and end-systole, were significantly higher in women with GDM (Table 2).

**Table 2:** Left ventricular parameters among women with normal pregnancies and gestational diabetes mellitus. Data are presented as mean ± SD. A p-value <0.001 compared to women with normal pregnancies. The independent t-test was used to calculate statistical differences. 2D: two-dimensional; E: early diastolic peak flow velocity; A: late diastolic peak flow velocity

Parameter	Normal pregnancies (n = 100)	Gestational diabetes (n = 100)	P-value	t-value
2D ventricular wall thickness, mm in end diastole	2.25 ± 0.36	2.95 ± 0.62	0.001	-9.76
2D ventricular wall thickness, mm in end systole	2.78 ± 0.57	3.56 ± 0.64	0.001	-9.09
Interventricular septum thickness, mm in end diastole	2.56 ± 0.53	3.56 ± 0.67	0.001	-11.71
Interventricular septum thickness, mm in end systole	3.95 ± 0.32	4.89 ± 0.99	0.001	-9.05
Fractional shortening	0.25 ± 0.11	0.34 ± 0.08	0.001	-6.62
E, cm/s	30.23 ± 3.5	34.24 ± 4.11	0.001	-7.41
E/A ratio	0.70 ± 0.11	0.67 ± 0.17	0.001	3.49

We also performed a multivariate analysis of GDM with left ventricular parameters adjusted for maternal factors (Table 3).

**Table 3:** Multivariate linear regression: association of GDM with left ventricular parameters adjusted for maternal factors. Note. Multivariate linear regression adjusted for maternal BMI, age, parity, gestational age at delivery, and birth weight. BMI: body mass index; CI: confidence interval; GDM: gestational diabetes mellitus; IVS: interventricular septum; ED: end diastole; ES: end systole

Parameter	β coefficient (GDM)	95% CI	P-value
2D posterior wall thickness (ED)	0.58	0.43 to 0.73	<0.001
2D posterior wall thickness (ES)	0.62	0.45 to 0.78	<0.001
IVS thickness (ED)	0.71	0.56 to 0.87	<0.001
IVS thickness (ES)	0.84	0.66 to 1.02	<0.001
Fractional shortening	0.09	0.05 to 0.13	<0.001
E-wave velocity (E)	3.21	2.43 to 4.00	<0.001
E/A ratio	-0.02	-0.05 to 0.01	0.18

No fetal cardiac anomalies were detected in either group, a finding confirmed through postnatal echocardiography during the neonatal period.

## Discussion

This study provides evidence that pregnancies complicated by GDM are associated with significant alterations in fetal cardiac dimensions and function compared to normal pregnancies. Notably, left ventricular posterior wall thickness and IVS thickness, both in diastole and systole, were significantly increased in the GDM group. These findings align with previous studies suggesting that maternal hyperglycemia influences fetal myocardial remodeling, likely mediated by fetal hyperinsulinemia and altered myocardial metabolism [8].

The observed increase in fetal cardiac wall thickness and IVS thickness in GDM pregnancies may be indicative of early myocardial hypertrophy, a phenomenon previously documented in infants of diabetic mothers. These structural changes could result from chronic exposure to elevated glucose levels and fetal hyperinsulinemia, which act as anabolic stimuli for myocardial cells. We observed impaired fetal cardiac diastolic function in pregnancies complicated by gestational diabetes, as evidenced by a reduced E/A ratio. This impairment may be attributed to factors such as increased ventricular wall thickness, decreased compliance, and altered ventricular contractility [9].

Research on the link between GDM and congenital heart defects has yielded inconsistent findings. The most commonly reported observation is a modest association between higher maternal BMI and the overall occurrence of congenital heart defects [10,11]. Conversely, fetuses of mothers with prior diabetes are well-documented to have a higher risk of congenital heart defects, along with neurological abnormalities [12-16]. Interestingly, despite the structural differences, we found no evidence of congenital heart anomalies in either group, which was confirmed by postnatal echocardiography. This underscores the fact that the cardiac changes in GDM pregnancies may not necessarily translate to overt congenital defects but could represent functional adaptations. The increased E velocity and altered E/A ratio in the GDM group further indicate potential diastolic dysfunction, which could predispose these fetuses to adverse cardiovascular outcomes later in life. In our study, the mean E/A ratio remained within gestational age-appropriate normative ranges (typically greater than 0.8 after 28 weeks), although it trended lower in fetuses with GDM. Subtle diastolic dysfunction may reflect early myocardial remodeling, but remains subclinical. Similarly, fractional shortening values, though preserved, should be interpreted with caution, given the limited sensitivity of fractional shortening in detecting early systolic dysfunction in fetuses.

The long-term effects of fetal cardiac structural changes on postnatal life remain uncertain. Recent evidence suggests that infants born to diabetic mothers may exhibit diastolic dysfunction. One study reported a prolonged deceleration time during early left ventricular diastolic filling, likely indicating impaired ventricular relaxation rather than reduced compliance [17,18].

Our study underscores the utility of fetal echocardiography as a screening tool in pregnancies affected by GDM, which may carry an elevated risk for subclinical fetal cardiac changes, even in the absence of other high-risk features. Routine fetal echocardiographic evaluations in GDM pregnancies could facilitate early detection of subtle cardiac alterations and guide clinical decision-making, including stricter glycemic control and tailored follow-up for at-risk neonates. Although HbA1c data were available, subgroup analysis based on glycemic control was limited by the small number of suboptimally controlled cases. Future studies with larger, more stratified samples are warranted to assess dose-response relationships between maternal glycemic control and fetal cardiac changes.

The lack of significant differences in birth weight and gestational age at delivery between the groups suggests that the observed cardiac changes may occur independently of gross fetal overgrowth or preterm birth, emphasizing the direct impact of maternal hyperglycemia on fetal myocardial development. However, it is important to note that these findings may vary with the severity and duration of GDM. Although this study identified subtle alterations in fetal cardiac function among GDM pregnancies, these findings should be interpreted as preliminary signals rather than definitive early markers of future cardiovascular risk. Longitudinal studies are needed to assess their predictive value over time.

Limitations

The relatively small sample size and single-center design may limit the generalizability of the results. Additionally, we did not stratify the GDM group based on glycemic control levels or insulin use, which could have provided further insights into the relationship between glycemic control and fetal cardiac changes. However, we used a standardized echocardiographic protocol performed by experienced operators, ensuring consistency and accuracy in measurements. A key limitation of our study is that although maternal BMI was not significantly different between groups, its potential confounding effect on fetal cardiac changes cannot be ruled out; however, multivariate analysis adjusting for BMI and other covariates confirmed that GDM remained independently associated with the observed alterations. The inclusion of postnatal echocardiographic confirmation adds robustness to the findings. An important limitation is the timing of recruitment. As GDM screening typically occurs between 24 and 28 weeks of gestation, women recruited at or before 20 weeks who later developed GDM may have been misclassified as non-GDM, potentially underestimating the differences between groups.

## Conclusions

Our study highlights the significant impact of GDM on fetal cardiac structure and function, emphasizing the presence of increased myocardial thickness and diastolic dysfunction in fetuses of mothers with GDM, even in the absence of congenital heart defects. These findings suggest that fetal myocardial remodeling begins in utero and may be an adaptive response to chronic maternal hyperglycemia. The observed changes, particularly the increased left ventricular posterior wall thickness, IVS thickness, and altered E/A ratio, may represent early signs of fetal cardiac hypertrophy and diastolic dysfunction, which could have implications for long-term cardiovascular health. The findings from our study support the role of routine fetal echocardiography as an essential tool in the prenatal assessment of GDM pregnancies. Early identification of these subclinical cardiac changes could provide an opportunity for targeted interventions, such as optimized glycemic control, tailored obstetric care, and close neonatal follow-up. Future research should focus on longitudinal studies that follow these infants beyond the neonatal period to determine whether these prenatal cardiac changes translate into long-term cardiovascular alterations, including an increased risk of hypertrophic cardiomyopathy, heart failure, or metabolic syndrome in later life. Expanding research in this field could lead to more personalized prenatal and postnatal care strategies aimed at mitigating long-term cardiovascular risks in children born to mothers with GDM.
